# Identification and characterization of an octameric PEG-protein conjugate system for intravitreal long-acting delivery to the back of the eye

**DOI:** 10.1371/journal.pone.0218613

**Published:** 2019-06-28

**Authors:** Whitney Shatz, Philip E. Hass, Nikhil Peer, Maciej T. Paluch, Craig Blanchette, Guanghui Han, Wendy Sandoval, Ashley Morando, Kelly M. Loyet, Vladimir Bantseev, Helen Booler, Susan Crowell, Amrita Kamath, Justin M. Scheer, Robert F. Kelley

**Affiliations:** 1 Protein Chemistry, Genentech Inc., South San Francisco, California, United States of America; 2 Microchemistry, Proteomics and Lipidomics, Genentech Inc., South San Francisco, California, United States of America; 3 Biochemical and Cellular Pharmacology, Genentech Inc., South San Francisco, California, United States of America; 4 Safety Assessment, Genentech Inc., South San Francisco, California, United States of America; 5 Pre-clinical and Translational Pharmacokinetics, Genentech Inc., South San Francisco, California, United States of America; 6 Drug Delivery, Genentech, South San Francisco, California, United States of America; University of Tasmania, AUSTRALIA

## Abstract

Innovative protein engineering and chemical conjugation technologies have yielded an impressive number of drug candidates in clinical development including >80 antibody drug conjugates, >60 bispecific antibodies, >35 Fc-fusion proteins and >10 immuno-cytokines. Despite these innovations, technological advances are needed to address unmet medical needs with new pharmacological mechanisms. Age-related eye diseases are among the most common causes of blindness and poor vision in the world. Many such diseases affect the back of the eye, where the inaccessibility of the site of action necessitates therapeutic delivery via intravitreal (IVT) injection. Treatments administered via this route typically have vitreal half-lives <10 days in humans, requiring frequent administration. Since IVT injection is burdensome to patients, there exists a strong need to develop therapeutics with prolonged residence time in the eye. We report here a strategy to increase retention of a therapeutic fragment antibody (Fab) in the eye, using an anti-complement factor D Fab previously optimized for ocular delivery. Polyethylene glycol structures, varying in length, geometry and degree of branching, were coupled to the Fab via maleimide-activated termini. A screening strategy was developed to allow for key determinants of ocular half-life to be measured in vitro. After compound selection, a scalable process was established to enable tolerability and pharmacokinetic studies in cynomolgus monkeys, demonstrating an increase in vitreal half-life with no associated adverse events. Further, we show that the technique for compound selection, analytical characterization, and scalable production is general for a range of antibody fragments. The application of the technology has broad impact in across many therapeutic areas with the first major advancement in the treatment of an important ocular disease.

## Introduction

Age-related eye diseases (AREDs) are among the most common causes of blindness and poor vision in the world [1,2]. Over the last 15 years, peptides, receptor domain fusions and antibody fragments (Fabs) have demonstrated efficacy as therapeutics for the treatment of AREDs such as neovascular (wet) age-related macular degeneration (nAMD), retinal vein occlusion (RVO) and diabetic macular edema (DME) among others [3,4]. However, delivery of these therapeutics to the back of the eye is often challenging owing to a multitude of factors, including inaccessibility of the site of action, restrictions on volume of injection (≤100 μL), a short list of formulation excipients compatible with ocular use [5], and a limited understanding of the mechanism of clearance from the eye [6,7]. Moreover, poor accessibility of the site of action necessitates administration of available treatments by injection into neighboring compartments such as into the vitreous, referred to as intravitreal (IVT) injection, currently the standard of care [8].

Because of the relatively short residence time of biological therapeutics and other small molecule therapies, maximal clinical benefit for treatment of retinal disorders is currently obtained with IVT injections at 4–8 week intervals [2]. However, frequent office visits impose a significant burden on patients, caregivers, physicians and the healthcare system. According to Ehlken et al., treatment and follow-up regimens are common problems in the management of patients with nAMD and DME and limit clinical treatment outcomes under real-life conditions [9]. In the case of anti-VEGF treatment, research suggests that suboptimal vision outcomes in patients with nAMD may be due to undertreatment [10–14]. Reducing the frequency of IVT injection can positively impact standard of care by increasing compliance, reducing frequency of office visits, as well as the incidence of injection-related complications, while maintaining optimal vision outcomes. Long acting delivery (LAD) technologies, including molecules with increased vitreal half-life, have the potential to improve patient quality of life. Since AREDs are prominent in the elderly population, often with either limited mobility and/or resource, and since loss of sight is such a debilitating condition, long-lasting back of the eye treatments that prevent blindness are considered an unmet medical need [2].

Previously, we used a pharmacokinetic (PK) rabbit model to explore molecular attributes of full-length antibodies and antibody fragments (Fabs) affecting vitreal half-life [15]. Our results demonstrated that in contrast to systemic clearance, FcRn-dependent recycling does not contribute to the vitreal half-life increase of IgG [15]. Using non-branched, single-site polyethylene glycol (PEG) + Fab conjugates, we further showed that diffusion and molecular size, parameterized as hydrodynamic radius (R_H_), are key contributors to the rate of vitreal clearance [16].

PEG has a long history of being used in the fields of biotechnology and pharmacology due to its many beneficial effects on molecule properties, such as by increasing solubility [17], half-life extension [18–20], or by decreasing immunogenicity [21]. However more recently, many novel PEG geometries with varied chain length, polydispersity and branching have become readily available, creating opportunities for the development of new therapeutics with quality attributes desirable for ocular IVT long-acting delivery.

Here, PEG scaffolds were conjugated to an anti-factor D Fab therapeutic (AFD.v14), previously engineered for physical and chemical stability in ocular compartments [22]. AFD is a Fab of a humanized immunoglobulin G1 (IgG1) monoclonal antibody directed against complement factor D (CFD), a highly specific chymotrypsin-like serine protease that is a rate-limiting enzyme in the activation of the alternative complement pathway (ACP) [23]. Although the prototype anti-CFD antibody Fab (AFD; FCFD4514S, lampalizumab) [24,25] did not meet the primary endpoint in a phase III clinical trial for geographic atrophy (GA), an advanced form of AMD, the dose (10 mg IVT) and frequency of injection served as guideposts for our conjugate design. Combining PK and formulation attributes suitable for long-acting delivery (LAD) to the back of the eye facilitated identification of a novel ocular IVT injectable system.

**Fig 1:**
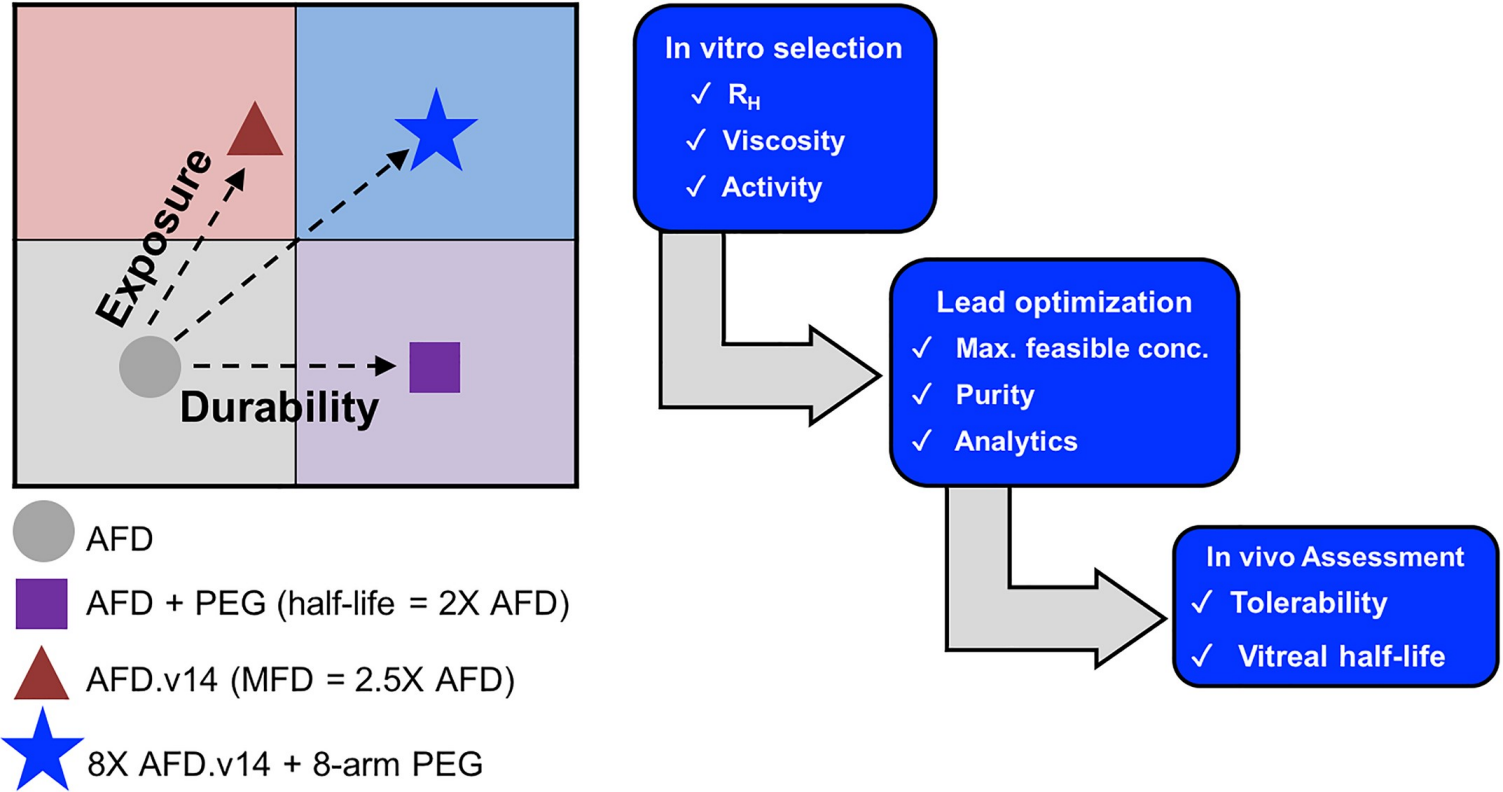
**A.** Schematic of the workflow process for selection, characterization and PK analysis of the novel Fab + PEG LAD system. **B.** By combining a Fab with improved formulation properties with a multivalent PEG scaffold, we were able to identify a next generation AFD.v14 + LAD with enhanced durability (x-axis) and exposure (y-axis) relative to AFD.v14 Fab alone.

In this study, we established a multi-tiered approach to develop a Fab + PEG conjugate with increased durability and exposure (Fig 1). Hydrodynamic radius (R_H_), ratio of carrier to active ingredient (AI), target binding and viscosity at the maximum feasible concentration (MFC) were all criteria evaluated for candidate selection (Fig 1A). Moreover, since protein + polymer complexes can be difficult to assemble and characterize, an accompanying biophysical toolbox capable of in-depth analysis of complex conjugation mixtures was also developed. Finally, our lead LAD candidate was compared to the Fab alone in a non-human primate (NHP) *in vivo* model, demonstrating both favorable safety and PK properties. By combining improved solubility and physical and chemical stability with an increase in R_H_, we describe herein a conjugate system that allows for gains in both exposure and durability (Fig 1B), yielding the potential to dramatically increase time between doses, while also better protecting vision.

## Results

### Ranking of Fab-PEG conjugates

**Fig 2:**
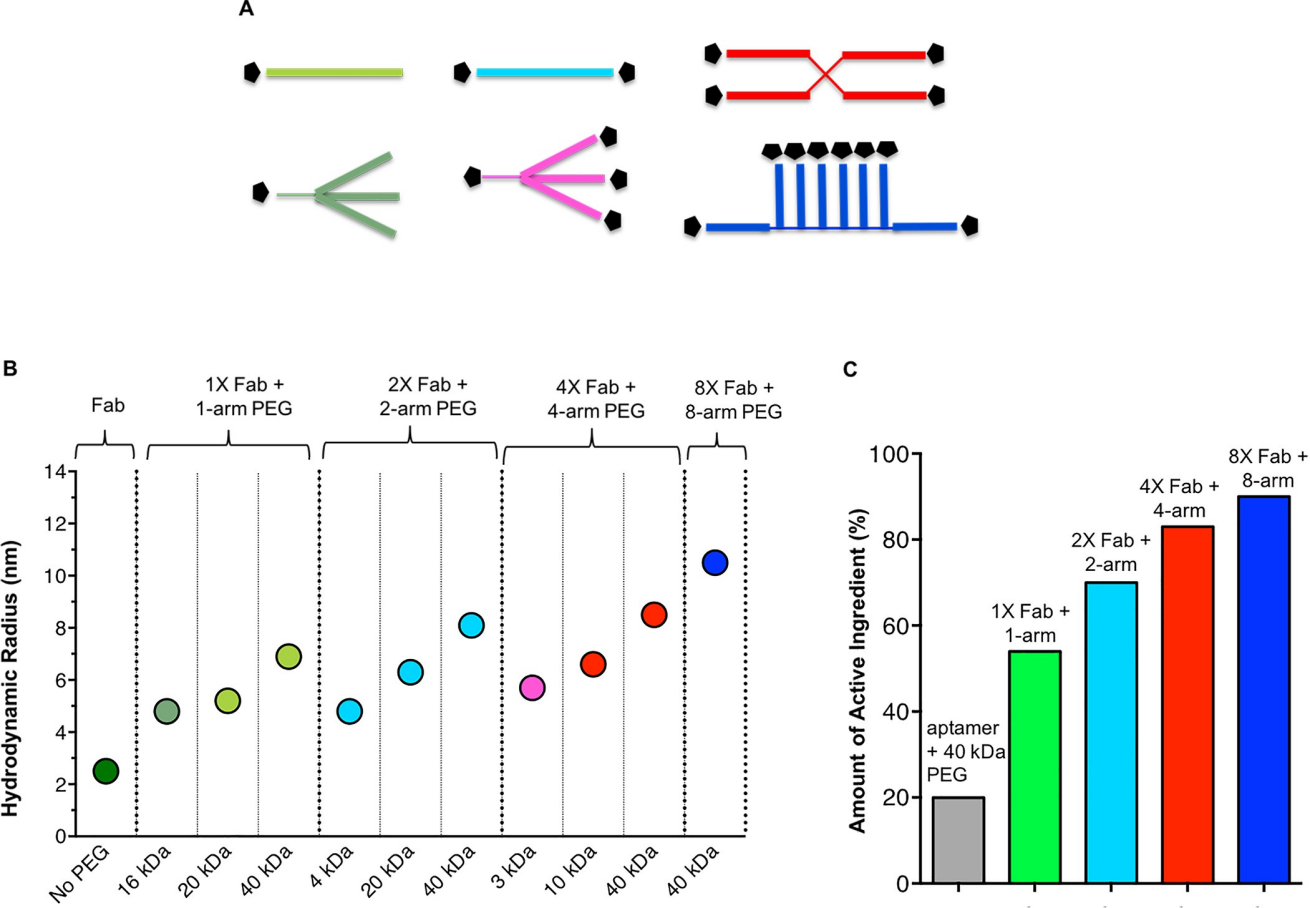
**A.** Schematic representation of the types of PEGs used. **B.** R_H_ values were used to rank the Fab + PEG conjugates. The conjugates are organized first in ascending degree of branching, and in increasing MW within each group. **C.** Increasing degree of PEG branching maximizes Fab content and decreases PEG burden in the eye. 40 kDa 4-arm and 8-arm PEG molecules increase R_H_ beyond 40 kDa linear PEG, while also decreasing PEG burden. Pegaptanib (Macugen) is an aptamer conjugated to 40 kDa PEG.

PEG structures terminated with either one maleimide, two maleimides, four maleimides or eight maleimides were coupled to AFD.v14 via a c-terminal cysteine on the Fab, resulting in ten unique Fab + PEG conjugates with varying chain length, geometry and number of reactive groups (Fig 2A and Table 1). The graph in Fig 2B displays measured R_H_ values (y-axis) for the ten different conjugates, clustered based on number of maleimide-activated PEG chains per structure, and organized in ascending MW within each grouping (x-axis). Without PEG, the R_H_ of the AFD.v14 is 2.5 nm, and its MW is around 50 kDa, while the ten different Fab + PEG conjugates yield radii ranging from around 5 to 10 nm, and molar masses between 63 kDa and 440 kDa. It is interesting to note that increasing the MW of the PEG portion of the Fab + PEG conjugates had a greater impact on R_H_ than increasing the number of conjugated Fabs. To better understand the relative contributions of the Fab and the PEG to the overall increase in R_H_, two data sets were extracted for further analysis (S2 Fig).

**Table 1:** List of the ten different PEG compounds that were used to make PEG+Fab conjugates. Specifications were determined by respective vendors. In some cases, substitution was not available as denoted by N/A.

Vendor	Catalog Name	MW (Da)	No. Arms	Substitution (%)
**Quanta Biodesign**	CAS # 11487	15,592.5	1	N/A
**NOF**	SUNBRIGHT ME-200MA	20,841	1	91.2
	SUNBRIGHT ME-400MA	41,182	1	93.4
	SUNBRIGHT DE-200MA	21233	2	88.0
	SUNBRIGHT DE-400MA	41,750	2	77.6
**Quanta Biodesign**	CAS # 11433	3,121.5	4	N/A
**NOF**	SUNBRIGHT PTE-100MA	11,035	4	94.6
	SUNBRIGHT PTE-400MA	41,505	4	92.9
	SUNBRIGHT HGEO-400MA	45,237	8	86.1
**JenKem**	8ARM(TP)-MAL-40K	39,609	8	95.2

Top candidates (four largest R_H_s) were also ranked based on the proportion of AI in each formulation. As shown in Fig 2C, the proportion of PEG carrier diminishes with increasing degree of branching, ranging from 54% AI when conjugated to the 1-arm PEG, to 90% when conjugated to the 8-arm PEG. From this panel, two Fab + PEG conjugates emerged as candidates with a greater than 3-fold increase in R_H_ relative to Fab alone. Both the 40 kDa 4-arm (10 kDa per arm) and the 40 kDa 8-arm (5 kDa per arm) had the additional advantage of contributing very little to the overall conjugate mass. As demonstrated in the analysis (Fig 2C), the 8-arm formulation contains 3 times less PEG mass than is present in pegaptanib sodium (1.6 mg dose, 20% AI), which is approved for the treatment of nAMD [16] and is also administered via IVT injection.

### Large scale production and characterization of lead molecules

**Fig 3:**
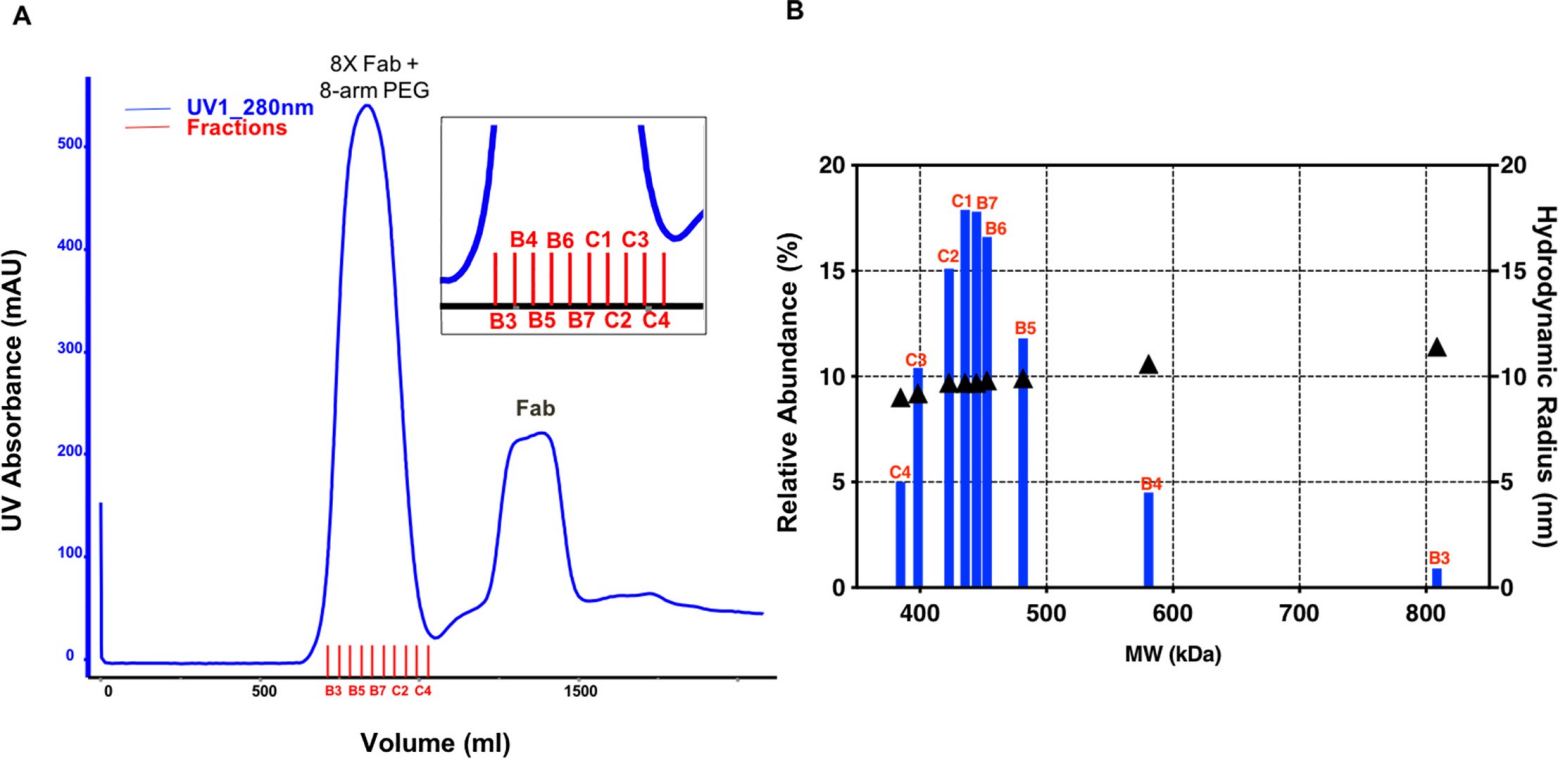
**A.** Example of purification chromatogram for scale-up production of 8-arm conjugate. An S-400 column was used to isolate each conjugation mixture and remove unreacted species. **B.** Fractions across the main peak were analyzed, by SEC-MALS to determine MW (x-axis) and by SEC-QELS (right y-axis) to determine R_H_. Additionally, these values were compared to each other based on their relative abundance (left y-axis). Analysis of the conjugation product reveals a gaussian distribution of MWs, with the greatest abundance centered around the desired specification based on Table 2. It is interesting to note that the measured R_H_ values do not change appreciably despite a wide range of MWs.

Each reaction pool was passed over an S-400 column to remove impurities and unreacted species, and to buffer exchange into phosphate buffered saline (PBS) pH 7.2. Fig 3A is an example of the SEC chromatograms that were generated for each conjugate, which shows the UV trace for conjugation of Fab to the 8-arm PEG at a Fab to maleimide ratio of 1.5 to 1. There are two prominent peaks and some lower abundance impurities. According to SEC-MALS measurements, the first peak is the Fab + PEG conjugated material with an average MW of 367.1 kDa and the second main peak is the unreacted Fab with an average MW of 49.6 kDa. The Fab pre-peak is composed of covalently crosslinked Fab species, while the post peak is likely composed of impurities from the PEG, though the scattering intensities for this peak was too weak to obtain accurate molar mass measurements. The mass range for the Fab + PEG conjugate was also observed by high-mass matrix-assisted laser desorption ionization (HM-MALDI), as shown in S1A Fig. In this spectrum, both singly (+1) and doubly (+2) charged states are detected, where visible peaks in between the assigned masses are due to loss of non-covalently attached light chain from the conjugated Fabs. A comparison between theoretical and measured MWs, as well as the average number of Fabs on each scaffold are shown in Table 2. While it is reasonable to assume that all reactive maleimides have been depleted during the reaction, the SEC-MALS results reveal that for both conjugates, the average number of Fabs per PEG scaffold are slightly below target (4 and 8 Fabs per PEG). Preliminary evidence suggested that this may be the product of heterogeneity within the branched PEG scaffolds, (S1B Fig) likely due to the absence of reactive maleimides.

**Table 2:** Comparison of theoretical MW of each conjugate relative to that measured by SEC-MALS. Average Fabs per PEG scaffold was determined by subtracting the MW of the PEG core and dividing by the MW of the Fab.

Molecule	PEG Core MW(kDa)	PEG Arm MW (kDa)	Theoretical MW (kDa)	Measured MW (kDa)	Average Fabs/ PEG Scaffold
**4X Fab + 4-arm PEG**	40	10	228.0	215.2 ±0.4	3.7
**8X Fab + 8-arm PEG**	40	5	416.1	367.5 ±0.7	7.0

Though the conjugate peak appears symmetrical, analysis of fractions across the main peak (B3-C4) revealed a more complicated mixture. In Fig 3B, the MW of each fraction (x-axis) is plotted against its abundance relative to the total mixture (left y-axis). Since polydispersity is inherent to PEG production, it is not surprising that MWs varied 2-fold across the fractionated peak. Nonetheless, the most abundant fractions (~20%) in the conjugation mixture are those centered around the target MW (416.1 kDa). Interestingly, differences in corresponding R_H_s for each fraction were modest, ranging from an average of 9 nm to 11.4 nm (right y-axis). Thus, for both 4-arm and 8-arm PEG conjugations, the choice was made to pool fractions within 5% of the target MW.

**Fig 4:**
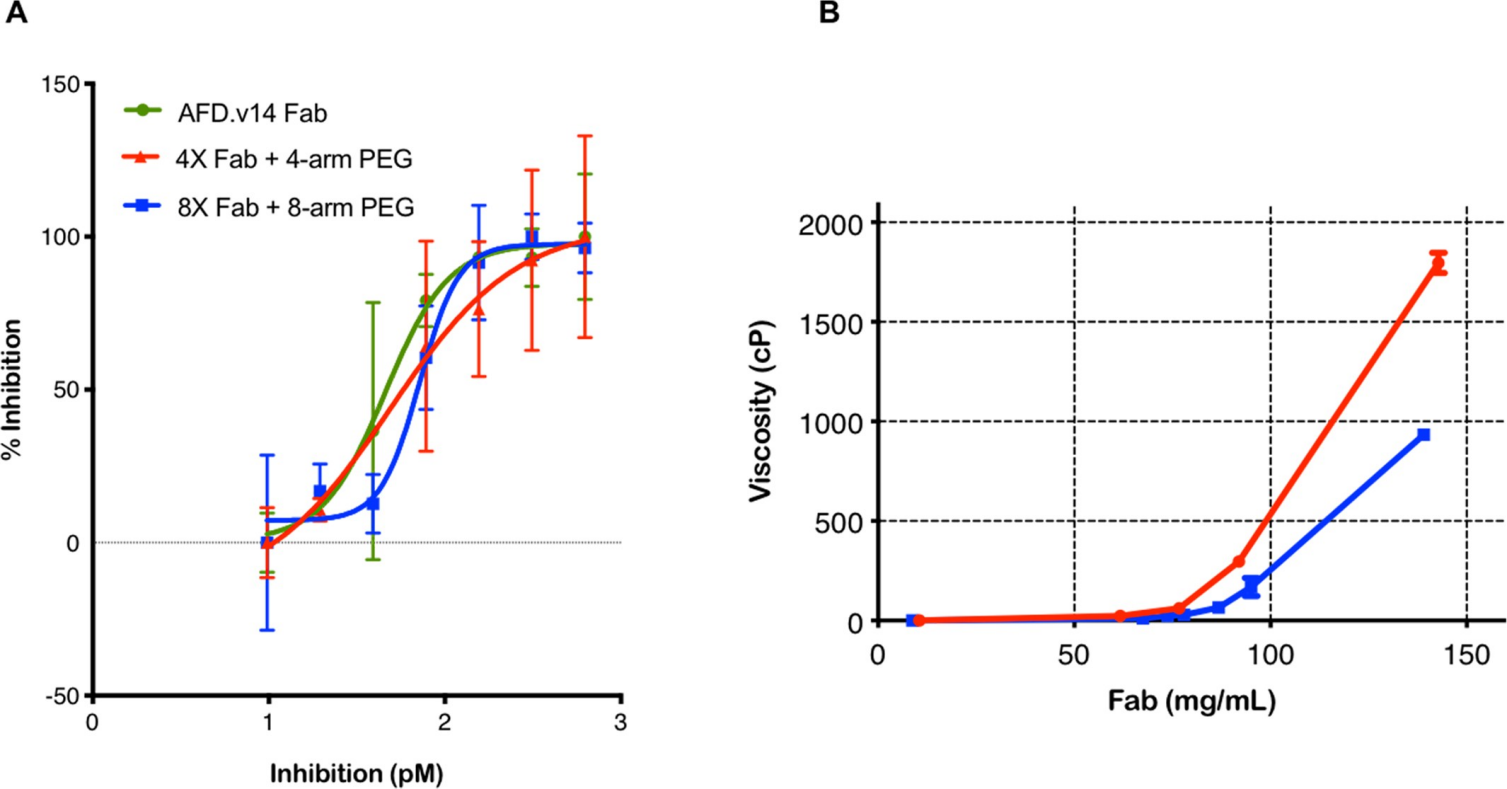
**A.** Fab alone, 4X Fab + 4-arm PEG and 8X Fab + 8-arm PEG conjugates all exhibited very similar behavior in the TR-FRET assay on a milligram of Fab basis. **B**. Visco-elastic behavior of 8X Fab + 8-arm and 4X Fab-4-arm PEG conjugates were compared at a sheer rate of 1/1000 seconds, in PBS at 25°C. The 8-arm formulation is significantly lower in viscosity than the 4X Fab + 4-arm PEG, at Fab concentrations above 70 mg/mL, as seen in the zoomed-in view.

A CFD-dependent cleavage of complement factor B assay was used to compare the inhibitory properties of the free and conjugated Fabs (Fig 4A). CFD is a monomeric target with a single binding domain for AFD Fab, thus inhibition curves were compared on the basis of Fab molarity using the Fab instead of PEG-Fab multimer MW for calculation. Indeed, the results in Fig 4A demonstrate similar activities, indicating no steric effects due to conjugation.

Viscosity of both the 4X Fab + 4-arm PEG conjugate and 8X Fab + 8-arm PEG conjugate were measured at Fab concentrations ranging between 8 and 143 mg/mL, with solution viscosity ranging between 1 and 2 x 10^3^ centipoise (cP). Overall, both conjugates exhibit similar behavior below Fab concentrations of 75 mg/mL (Fig 4B). Around this concentration, the slope of the two curves begin to diverge, revealing a clear advantage of the 8-arm PEG conjugate over the 4-arm version for formulation at high concentrations. For this reason and because of the decreased PEG burden, the 8-arm PEG conjugate was chosen as the lead for the NHP safety and PK studies. Once the lead PEG scaffold was established, the system was additionally evaluated by conjugating to three different Fabs. Assessment by SEC-QELS (S3A Fig) and SDS-PAGE gel (S3B Fig), yielded very similar profiles, demonstrating that this approach is robust, reproducible and broadly applicable.

**Fig 5:**
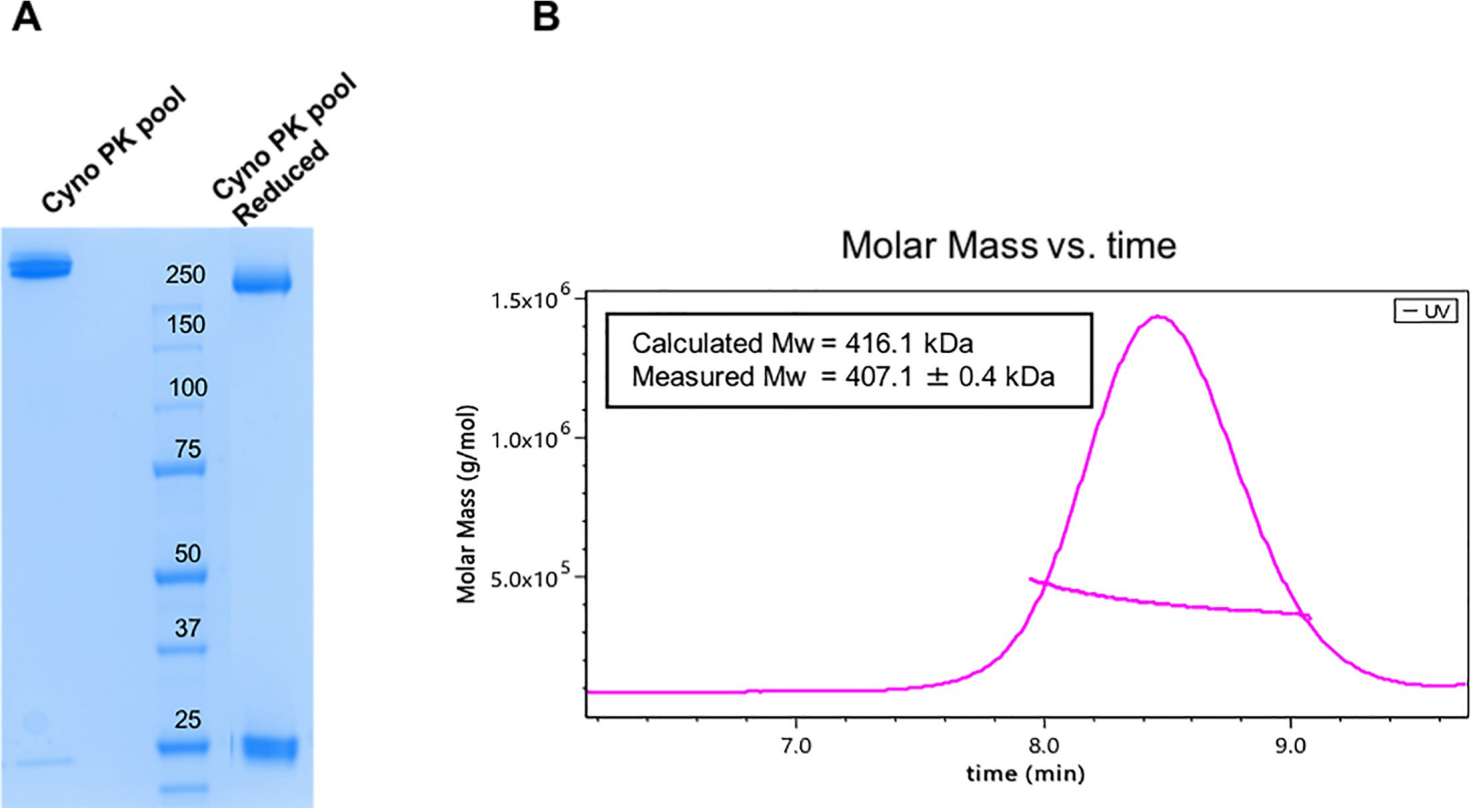
**A.** SDS-PAGE of final material, where the left lane is loaded with non-reduced Fab-PEG conjugate while the right lane contains the addition of DTT to reduce the interchain disulfide between HC and LC. Both lanes of the gel demonstrate a very tight band, with a high degree of purity, and little visible contaminants. **B.** SEC-QELS for 8X Fab + 8-arm PEG conjugate, with measured R_H_ across UV trace demonstrates reproducibility independent of Fab identity.

The purification scheme for the 8X Fab + 8-arm PEG conjugate was further refined to include a cation exchange (CEX) chromatography step, using a very shallow gradient elution to enable removal of trace level impurities (S4A Fig). Though complete resolution was not evident by UV, SDS-PAGE gel analysis of fractions collected across the main peak (S4B Fig) revealed the additional chromatography step was successful in separating heterogeneity still present in the formulation and in enriching for the target product. Characterization of the final pool (fractions 3,4,5 concentrated to 39 mg/mL) demonstrated a discrete band, both with and without reducing agent, little visible contaminants by SDS-PAGE (Fig 5A), and little variation in MW by SEC-MALS (Fig 5B).

### Nonclinical *in vivo* Safety Assessment

Single dose IVT administrations in cynomolgus (cyno) monkeys of saline, an N-ethylmaleimide capped 8-arm capped PEG and 8X Fab + 8-arm PEG at doses of 8.1 and 13.4 mg/mL Fab (Table 3) with 29-day follow up, were well tolerated on clinical ophthalmic examination up to the highest dose tested. White vitreous floaters which may have represented test article, were observed during ophthalmic examination in eyes administered 8X Fab+8-arm PEG at both dose levels. The incidence, duration and size of these vitreous opacities followed a dose-response pattern such that the vitreous floaters were larger, more frequent and more persistent in eyes administered the highest dose of 8X Fab + 8-arm PEG.

**Table 3:** NHP tolerability (Tol.) and PK study details. Dosing was performed via bilateral IVT injection of 0.1 mL dosing solution per eye. Pegaptanib was included for reference where N/A denotes “not applicable”.

Study Type	Group #	Molecule	Dose/Eye (mg)	AI/Eye (mg)	PEG/Eye (mg)	Animals/ Group
**Tol.**	1	Saline control	0	0	0	2
	2	8-arm capped PEG	1.4	0	1.4	2
	3	8X Fab + 8-arm PEG	8.1	7.1	1.0	3
	4		13.4	11.8	1.6	3
**PK**	1	Fab	6.6	6.6	0	4
	2	8X Fab + 8-arm PEG	4.3	3.9	0.4	10
	3		1.3	1.2	0.1	10
N/A	N/A	pegaptanib	0.3	0.12	0.18	N/A

**Fig 6:**
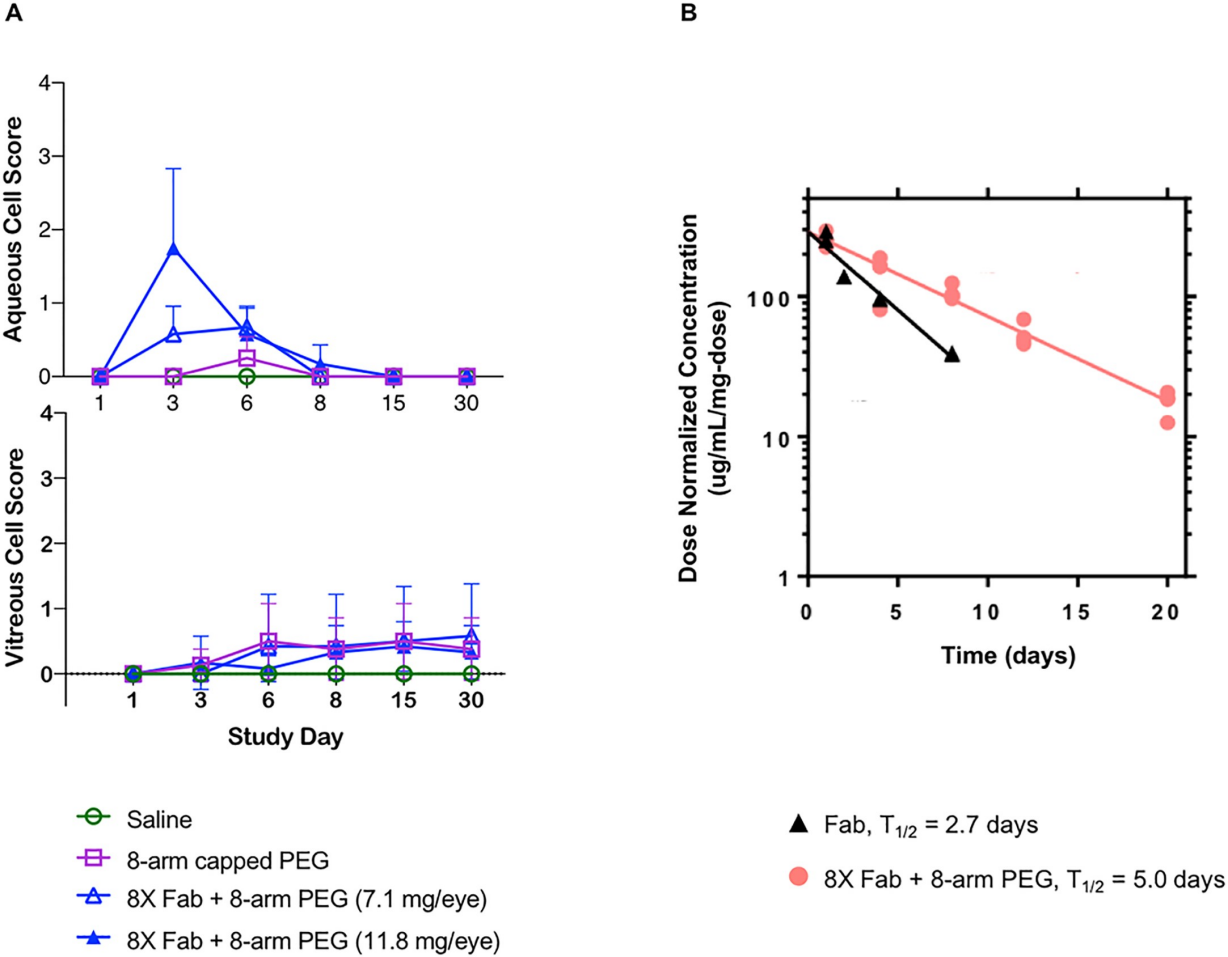
**A.** Aqueous (top) and vitreous (bottom) cell scores were measured in monkeys after a single administered dose. Transient anterior and posterior segment ocular inflammation was noted following a single dose IVT administration of PEGylated drug. Aqueous cell peaked on day 3 rapidly improving and completely resolving by day 8. In contrast to the anterior segment, a posterior segment inflammatory response (characterized by vitreous cell) increased to mild until day 6 and decreased to Trace levels that persisted until the last examination on day 30. Data are reported as the mean SD; Trace findings were assigned a value of 0.5. **B.** 8X Fab + 8-arm PEG vs. Fab PK in monkeys following intravitreal administration. Fab had a vitreal half-life of 2.7 days, whereas the 8X Fab + 8-arm PEG at top dose had increased half-life of 5 days.

Serum exposure for 8X Fab + 8-arm PEG was approximately dose proportional (Table 4) and treatments had very little effects on clinical observations, body weights, intraocular pressure, retinal abnormalities via spectral domain optical coherence tomography (sdOCT), or macroscopic necropsy observations (S4 and S5 Figs). A post-dose transient anterior segment inflammatory response (characterized by aqueous cell and aqueous flare) was observed in eyes administered each test article and control article (Fig 6A top panel) but the inflammatory response resolved in all eyes by Day 8. A posterior segment inflammatory response (characterized by vitreous cell) was also noted in eyes administered with each test article and control article (Fig 6A bottom panel), but in contrast to the anterior segment response, this posterior segment inflammation persisted until the last examination on Day 30. The greatest severity of vitreous cell was noted in animals administered 8.1 mg/eye 8X Fab + 8-arm PEG (Group 3).

**Table 4:** NHP serum results for 8X Fab + 8-arm PEG. Toxicokinetic parameters are reported on the basis of active ingredient (Fab) content.

Group #	Molecule	Dose/Eye (mg)	C_max_ (μg/mL)	AUC_all_ (μg/mL*day)	t_max_ (day)
**3**	8X Fab + 8-arm PEG	8.1	29 ± 14	290 ± 130	7
**4**		13.4	51 ± 9	620 ± 330	1.67

### Monkey PK confirmed half-life improvement

Following safety assessment, a monkey PK study was performed with single, bilateral IVT dose administration (Table 3). Vitreous concentration-time profiles of Fab and 8X Fab + 8-arm PEG at two different concentrations are presented in Fig 6B and pharmacokinetic parameters are presented in Table 5. Clearance from vitreous humor was consistent with first order kinetics for both AFD.v14 Fab and Fab + PEG. Similar to other Fabs [26] the non-conjugated Fab had a vitreal half-life of 2.7 days, whereas the 8X Fab + 8-arm PEG had an increased half-life of 4.2 ± 1.1 days (Table 5 and Fig 6B); dose normalized exposure was approximately two-fold higher for octamer (2120 ± 17 **μ**g/mL*day/mg Fab) vs. Fab (980 **μ**g/mL*day/mg). Vitreal exposure for octamer dosed at 1.3 or 4.3 mg/eye (1.1 or 3.9 mg Fab/eye) was dose proportional on the basis of AUC (2140 **μ**g/mL*day/mg Fab vs. 2110 **μ**g/mL*day/mg Fab); however, a higher dose-normalized C_max_ (310 **μ**g/mL/mg vs. 250 **μ**g/mL/mg) and lower volume of distribution (3.1 mL vs. 3.4 mL) were observed for the 1.3 mg/eye dose group relative to the 4.3 mg/eye dose group.

**Table 5:** PK parameters for Fab and 8X Fab + 8-arm PEG in NHP vitreous. Pharmacokinetic parameters are reported on the basis of AI (Fab) content.

Group #	Molecule	Dose/Eye (mg)	C_max_ (μg/mL)	AUC_inf_ (μg/mL*day)	t_1/2_ (day)	Cl (mL/day)	Vz (mL)
**1**	Fab	6.6	1380 ± 105	6450	2.7	0.79	3.1
**2**	8X Fab + 8-arm PEG	4.3	990 ± 55	8220	5	0.47	3.4
**3**		1.3	380 ± 11	2570	3.5	0.47	3.1

## Discussion

We have developed a novel, multimeric Fab + PEG soluble system, administrable by IVT injection, which has yielded an increase in vitreous half-life and favorable ocular tolerability in NHP *in vivo* studies. By demonstrating this relationship in a primate species, these results corroborate and broaden a previously reported hypothesis that diffusion is a significant driver of ocular elimination [26], now within the context of a preclinical model species with ocular anatomy and physiology more relevant to human. These findings support a novel platform for delivery of therapeutics to the back of the eye, with the potential for every other month dosing in humans, resulting in fewer IVT injections per year than monthly dosing of non-conjugated Fab.

Conjugation to PEG has been widely employed for systemic half-life extension and is generally considered safe for use in a wide variety of settings [26]. The recent availability of multimeric PEG structures has yielded new insights into the influence of PEG on the biophysical and biochemical properties of a Fab. Though it is not surprising that both polymer and protein impact R_H_, it is interesting to note that their relative contributions to overall size differ per unit of mass (Fig 2B). For example, the R_H_ of two Fabs conjugated to a 20 kDa PEG was smaller than one Fab conjugated to a 40 kDa PEG even though the mass of the former is greater than the latter. Moreover, two Fabs conjugated to a 40 kDa dimeric PEG had an R_H_ comparable to that of four Fabs conjugated to a 40 kDa tetrameric PEG. Comparing the 1-arm 40kDa PEG-Fab conjugate to the 40 kDa PEG-octamer conjugate suggests that Fab makes a non-additive contribution to R_H_, where 7 Fabs only increased the R_H_ by about 3 nm. The intuitive explanation is that the Fab has a globular structure whereas the PEG chain is likely in an extended structure with larger effective hydrodynamic volume, and thus a dominant contributor to R_H_ within the conjugates. Overall, the 8-arm PEG conjugate was the preferred molecule not only for its high R_H_ but also because the higher AI% delivered more protein with less carrier per injection.

Since IVT drug delivery is limited by the maximal volume of injection (≤100 ul), a high concentration formulation is important for delivery of the maximum feasible dose. However, it has been shown that coupling a therapeutic Fab to PEG has the potential to increase viscosity by increasing the excluded volume of the Fab therapeutic at high concentrations [26]. Therefore, understanding visco-elastic properties of the top candidates at a range of formulation concentrations intended to enable high dose scenarios (>50 mg/mL) was of importance in their evaluation. Moreover as demonstrated by Zarzar et al., PEG geometry also has an impact the visco-elastic properties of the Fab [26]. Both the 4X Fab + 4-arm PEG and 8X Fab + 8-arm PEG conjugate formulations were able to achieve concentrations >100 mg/mL Fab; however, the visco-elastic behavior of these conjugates diverged at concentrations greater than 75 mg/ml. We hypothesize that the 8-arm PEG conjugate enables a lower viscosity profile than the 4-arm at higher concentrations because the Fab-Fab interaction appears to be attractive, whereas the PEG-PEG interaction is repulsive [26]. The higher AI% of the 8X Fab + 8-arm PEG as compared to the 4X Fab + 4-arm PEG ensures a higher proportion of Fab is dominating the intramolecular interactions. This higher Fab ratio may explain why a higher MFC (>100 mg/mL) can be achieved with lower viscosity than with 4X Fab + 4-arm PEG (Fig 4B). Investigating visco-elastic behavior was not only informative for understanding biophysical characteristics of the Fab + PEG conjugates, but was also key to understanding the developability of this approach. Restrictions on ocular injection volume (≤100 **μ**L) and needle bore (≤30 gauge) for IVT injection in humans can mean that for some targets high therapeutic concentrations are required. For this reason, high viscosity at the MFC can result in unacceptable injection force and also in challenges in manufacture of drug product.

Once the lead candidate was chosen, safety and PK were assessed in an *in vivo* ocular monkey model. A single bilateral IVT injection of PEG alone or 8X Fab+8-arm PEG was generally well tolerated for up to 30 days. Common findings to all of the articles included a transient anterior segment inflammatory response that resolved within one week of dose administration, an inflammatory response in the posterior segment persisting through Study Day 30, and mononuclear cell infiltrates in single or multiple ocular tissues. White in color vitreous floaters noted in the 8X Fab + 8-arm PEG-treated eyes on the slitlamp biomicroscopy clinical ophthalmic examination may have represented the drug and their location away from the visual axis did not pose any clinical concern. Overall, the ocular tolerability profile reported here is similar to AFD single dose IVT that was recently published [27–29], suggesting this molecule will be well tolerated in the clinic. Additionally, extension of Fab vitreal half-life was also confirmed in a PK monkey model (Fig 6B). Subsequent *in vivo* experiments of this and related therapeutic candidates across a range of dose levels have demonstrated that the observed variability in half-life of the octamer across the two dose levels was attributable to experimental variability rather than dose-dependent pharmacokinetic behavior [24,30]. However, despite the observed variability, the vitreal half-lives in both 8X Fab + 8-arm PEG groups are consistent with significant half-life extension relative to the range of half-lives reported for molecules with smaller hydrodynamic radii, such as Fab [24,25] or full length antibodies [31,32]. Consistent with previous observations in rabbit ocular studies [16], vitreal half-life in monkeys appears to show a linear dependence on hydrodynamic radius, supporting diffusion rate within the vitreous as a key contributor to clearance.

In conclusion, we have successfully adapted a commercially available branched PEG for use as an injectable LAD technology for delivery of therapeutics to the back of the eye. R_H_ and viscosity measurements were critical for in vitro evaluation of the conjugate systems. The value of the chosen Fab + PEG conjugate system was demonstrated based on its ease of production, reproducibility of high quality using various Fabs, process scalability and amenability to high concentration formulations. Though paired only with different Fabs, this system positions itself to be amenable to conjugation with a wide variety of thiol-containing therapeutics or moieties. In contrast to other LAD technologies such as sustained delivery devices or slow release formulations ([33]), an approach based on half-life increase through polymer conjugation can enable similar C_max_ levels as observed with monthly IVT injections of free antibody Fab. Although a slow release formulation such as a PLGA implant can provide an increase in duration of treatment, a limitation in protein therapeutic loading to about 10% [34] suggests that to deliver a 10 mg antibody Fab dose to the eye the implant would be too large to be injectable. Similarly, a sustained delivery device may require a surgical procedure for implantation and that is less desirable than an IVT injection that would be feasible with the 8-arm PEG conjugate.

By combining AFD.v14, Fab engineered for high dose formulation, with a PEG geometry enabling increased AI and increased R_H_, we established a system with demonstrated safety and increased exposure and durability, as confirmed in NHPs. The approximate 2-fold increased exposure should translate to a 2-fold reduction in dosing frequency in human relative to the non-conjugated antibody Fab. Though realization of this benefit awaits clinical evaluation, the technology described here has the potential to provide significant benefit to the intended patient population by reducing overall treatment burden in posterior eye diseases such as AMD.

## Materials and methods

### Selection and characterization of PEG starting material

PEGs were procured from Quanta Biodesign (CAS # 11487, 11433) NOF (SUNBRIGHT ME-200MA, SUNBRIGHT ME-400MA, SUNBRIGHT DE-200MA, SUNBRIGHT DE-400MA, SUNBRIGHT PTE-100MA, SUNBRIGHT PTE-400MA, SUNBRIGHT HGEO-400MA) and JenKem (SKU: A4010, SKU: A10020). PEGs were chosen for one of the following reasons: low polydispersity, high chain length, high degree of branching or purity and reactive end group.

### Construction, expression and fab purification

Proteins were expressed in *E*.*coli* cells and purified, according to the methods described previously [22,26]. Briefly, proprietary plasmids were transformed into *E*.*coli* strain 64B4, and cells were cultured in a 10 L biofermentor. Once harvested, cells were ruptured, spun down and the collected supernatant was passed over an affinity column. Protein was captured by immunoaffinity chromatography on Protein G- Sepharose (GE Healthcare) and eluted with a low pH buffer. A secondary polishing step was performed at pH 5.0, using cation exchange chromatography (SPHP, GE Healthcare) with sodium chloride (NaCl) gradient elution. Pooled fractions were concentrated to around 5 mg/mL using Amicon Ultra-15 spin concentrators with 10,000 molecular weight cutoff (MWCO).

### Conjugation and formulation

In all conjugations, Fab was coupled to PEG via covalent attachment of maleimide activated termini to the c-terminal cysteine of the antibody fragment as previously described [16,26]. Each PEG was dissolved in 25 mM sodium acetate, pH 5.0 to limit maleimide hydrolysis. Prior to each conjugation, the Fab pool was titrated to pH 6.5 using 1 M (4-(2-hydroxyethyl)-1-piperazineethanesulfonic acid) (HEPES) at pH 7.2. Protein and polymer were mixed together at 2X molar excess over reactive maleimide. Conjugation pools were held overnight at room temperature. Following conjugation, the protein-polymer pools were buffer exchanged using a preparative scale GE Healthcare S-400 column in 20 mM histidine acetate, 50 mM NaCl pH 5.5 and fractionated to remove unreacted Fab and species with incomplete conjugation as previously described [26]. When necessary, formulations were concentrated using Amicon Ultra-15 (30,000 MWCO) spin concentrators and filtered using a Nalgene 0.2 **μ**m filter.

### Analysis of PEG and Fab contributions to overall R_H_

Data sets were evaluated and linear regression were performed using Prism Graph Pad.

### Scale-up conjugation

Stoichiometry for scale-up production of the lead candidates was optimized by comparing three different Fab to PEG ratios (1.5X, 3X and 5X). Comparison of SEC-MALS results revealed that conjugation efficiencies were comparable using all three ratios, and that only a slight molar excess of Fab (1.5X) was sufficient to deplete reactive maleimide. The amount targeted for scale-up was chosen to be 500 mg of final product to enable enough for multiple rheological measurements at various high concentrations. In order to reduce waste of starting materials, Fab was conjugated to both the tetrameric PEG and octameric PEG at a molar excess of 1.5X, 3X or 5X over each reactive maleimide to determine optimal ratios for scale-up. Analysis of reaction efficiency was performed by passing each conjugation mixture over SEC-MALS and determining ratio yielding highest amount of desired product.

### Polishing step

A 5 mL prepacked SPHP column (GE Healthcare) was used to further isolate the desired product. The conjugate pool was titrated down to pH 5.0 using acetic acid and loaded onto the column. Buffer A, composed of 20 mM sodium acetate, pH 5.0 was used to wash away non-specific contaminants, while buffer B comprised of buffer A with the addition of 1M NaCl was employed for elution. A very shallow gradient from 0–20% buffer B, over 50 CVs was successful in isolating the 8x Fab + 8-arm PEG from the remaining impurities as demonstrated in S2B Fig.

### Characterization methods

#### RP-LCMS

Data was acquired as described before [26] using an Agilent 1290 Infinity UPLC in tandem with an Agilent 6230 electrospray ionization time-of-flight (UPLC-ESI/ TOF) mass spectrometer, operating in positive ion mode. Unconjugated protein was loaded onto a reverse phase (RP) PLRP-S column (Agilent) with dimension of 4.6 x 50 mm. Mobile phase A consisted of 0.05% trifluoroacetic acid (TFA) while mobile phase B consisted of 0.05% TFA and 80% acetonitrile (ACN), and was used for the gradient between 20–90% solvent B.

#### SDS-PAGE

As previously described [26], Prior to conjugation, purity of the Fab and the FabC (with C-terminal cysteine) was determined using 4–20% Tris-Gly sodium dodecyl sulfate polyacrylamide gel electrophoresis (Thermo Scientific Pierce) run at 250 mV for 40 min, while purity of the conjugated protein-polymer complexes was determined after the final chromatography step using a 4–12% Bis-Tris Plus SDS PAGE gel run at 250 mV for 1.5 hours (Thermo Scientific Scientific).

#### Multi-angle Light Scattering (MALS)

As previously described, conjugation efficiency, as well as final purity of each pool, was determined using a Shodex SB 804 OHpak analytical SEC column run on a Dionex UltiMate 3000 UPLC (Thermo Fisher Scientific), with isocratic gradient of phosphate buffered saline (PBS) with an additional 150 mM NaCl spiked in, coupled to a multi-angle light scattering system (SEC-MALS) from Wyatt Instruments, which determined molar mass and size of the Fab + PEG conjugates [26].

#### Quasi-elastic light scattering (QELS)

Diffusion coefficients (D) were measured using quasi elastic light scattering (QELS) where fluctuations in intensity of laser light scattered were captured using a single photon counting module detecting at a 99.0^o^ angle. Data collection and processing was generated under the control of a PC driven by Wyatt Technology Corporation ASTRA software. Assuming a spherical shape, the Stokes-Einstein relationship was used to calculate R_H_ from D.

#### Rheology

Viscosity measurements were made using the Anton Paar Physica MCR 501 rotary rheometer, with the CP20-0.5^o^ cone and plate configuration. The CP20-0.5 geometry has a diameter of 20-mm and an angle of with 0.5°. The measurements were performed at 25°C using the temperature controller of the rheometer (Peltier plate with circulating fluid from water bath). Approximately 20 **μ**L of each sample was loaded onto the bottom plate for measurement, after which the cone was lowered slowly to the desired gap width. The measured torque determined the shear stress, from which the viscosity was calculated, as previously described [26].

#### TR-FRET

Activity of Fab-PEG conjugates were assessed using a Factor B cleavage TR-FRET assay, as described in Tesar et al. [22]. The AFD Fab + PEG conjugate molarity was calculated using the Fab MW in lieu of the multimer MW due to the expected 1:1 stoichiometry of Factor D inhibition by Fab.

#### Mass spectrometry and high mass MALDI

PEG powder was reconstituted to 10mg/mL in water. 20mg/ml α-Cyano-4-hydroxycinnaminic acid matrix solution dissolved in 50% acetonitrile:0.1% trifluoroacetic acid was employed as MALDI matrix. Three **μ**L of PEG solution was mixed 1:1:1 with sodium trifluoroacetate and 50% acetonitrile:0.1% trifluoroacetic acid. One μL of the PEG mixture was deposited onto a MALDI plate. One μL of matrix was added to the same spot and allowed to dry at ambient temperature. Analysis was performed on a 4800 MALDI TOF/TOF instrument (Sciex) in linear mode with m/z range from 5000–100000 and a 200 Hz Nd:Yag laser power of 4000. Peaks were visualized using Data Explorer software (Sciex).

High Mass MALDI analysis of PEG-Fab conjugates also occurred on a 4800 MALDI TOF/TOF equipped with an HM4 high mass detection retrofit system (CovalX). Equal volumes of PEG-Fab conjugate and 20 mg/mL sinapinic acid matrix (in 50% acetonitrile:0.1% trifluoroacetic acid, Agilent) were deposited onto a MALDI target plate for analysis. The m/z range was set from 100000 to 500000, with target mass set to 400000. Spectra were visualized using Data Explorer then annotated manually.

### In vivo NHP studies

All procedures conducted in animals complied with the Animal Welfare Act, the Guide for the Care and Use of Laboratory Animals, and the Office of Laboratory Animal Welfare. Protocols were approved by the local Institutional Animal Care and Use Committee (IACUC, Covance Laboratories Inc.) and done in accordance with the Association for Research in Vision and Ophthalmology's Statement for the Use of Animals in Ophthalmology and Vision Research. Covance Laboratories is fully accredited by the Association for Assessment and Accreditation of Laboratory Animal Care (AAALAC). All procedures were in compliance with applicable animal welfare national, state and local laws and regulations, including the US Animal Welfare Act, the US Public Health Service Policy on Humane Care and Use of Laboratory Animals.

Naïve male cyno monkeys (Covance Research Products, Inc.) assigned to each intravitreal study were 33 to 44 months old and ranged in weight from 2.4 to 3.7 kg at study initiation. During acclimation and the test period, animals were socially housed in standard 4-bank housing, with up to three animals per cage, except during study-related events, where they were individually housed in stainless steel cages with the following dimensions: 0.61 m width, 0.69 m length and x 0.76 m height. Animals were housed in an environmentally controlled room, maintained at 20°C to 26°C, a relative humidity of 30% to 70%, a minimum of 10 air changes per hour and a 12-hour light/12-hour dark cycle. As another method of enrichment, toys were provided and rotated once a week in order to maintain the interest of the animals and increase usage. Food consisting of Certified Primate Diet #5048 (PMI Nutrition International Certified LabDiet), along with supplemental dietary enrichment, were dispensed two times daily and water was provided ad libitum.

At least twice daily, animals were observed for mortality and signs of distress. Assessment of tolerability was also based on clinical observations, qualitative food consumption, body weights, ophthalmic examinations (slit lamp biomicroscopy and indirect ophthalmoscopy), intraocular pressure (IOP) measurements, spectral domain optical coherence tomography (sdOCT), and clinical and anatomic pathology.

Prior to the IVT injections, animals were anesthetized with intramuscular injections of midazolam (0.2 mg/kg) ketamine (5 mg/kg), and dexmedetomidine (0.025 mg/kg). A topical anesthetic (0.5% proparacaine) was instilled in each eye before the dose administration. Test articles were formulated in PBS pH 7.2, protein concentrations denoted in Table 3, with endotoxin levels below 0.1 EU/eye for all groups. Doses were administered by a board-certified veterinary ophthalmologist, using sterile insulin syringes filled under a laminar flow hood immediately prior to dosing. Following aseptic preparation of the ocular surface, each dose was administered as a 2 x 50**μ**l injection approximately 15 minutes apart. A topical antibiotic (Tobrex) was instilled in each eye following dosing. On Day 1, prior to IVT dosing and 6 hours post-administration, tramadol was administered orally. Upon recovery from anesthesia, buprenorphine SR, a sustained-release semi synthetic opioid, was administered by subcutaneous injection to provide analgesia. Additionally, a bland ophthalmic ointment (e.g., Lubrifresh or equivalent) was applied post dose as needed. Eyes were dosed in the infero-temporal quadrants through the pars plana. Following injection, eyes were examined by slit lamp biomicroscopy and/or indirect ophthalmoscopy.

#### IVT ocular tolerability

Male monkeys were randomly assigned into groups and received IVT doses bilateral IVT injections of vehicle, 8-arm PEG vehicle control or 8X Fab + 8-arm PEG (Table 3). Blood samples were collected for pharmacokinetic, anti-drug antibody evaluations and alternative pathway analysis. Approximately 1 mL of blood was collected from the femoral vein at 0.25, 1,6, 24 (Study Day 2), 48 (Study Day 3), 72 (Study Day 4), 96 (Study Day 5), 120 (Study Day 6), 168 (Study Day 8) and 240 (Study Day 11) hours post dose as applicable.

On Day 30 animals were euthanized via overdose of sodium pentobarbital. At necropsy, an examination of the external features of the carcass; external body orifices; abdominal, thoracic, and cranial cavities; organs; and tissues was performed. A selected set of tissues was collected and processed for hematoxylin and eosin (H&E) staining and subsequent microscopic analysis. Ocular tissues were embedded and sectioned to facilitate examination of the injection site, optic disc, and optic nerve. Sectioning of the eye included a cross section of the optic nerve in addition to the standard section. Sections were analyzed by a board-certified veterinary anatomic pathologist.

#### IVT PK assessment of Fab and 8X Fab + 8-arm PEG clearance from cyno vitreous

Naïve cyno monkeys were assigned to dose groups and dosed with test items at Covance Laboratories and observed for up to 29 days post-injection.

Ocular tissues were harvested only at terminal collections, at 1, 4, 8, 12, and/or 20 days post-injection, for analysis of test article concentration. Animals were euthanized via overdose of sodium pentobarbital, and blood collected via cardiac puncture to facilitate ocular tissue collection. At the time of euthanasia, both eyes were enucleated. Aqueous humor was collected with a sterile syringe, and each eye frozen in liquid nitrogen for 15–20 sec and placed on dry ice. Within one day, frozen vitreous humor was collected and stored at -70°C until analysis.

A Gyrolab XP assay was used to quantify Fab concentrations in cyno monkey vitreous humor. Samples were diluted 1:4 in sample buffer (PBS pH 7.4, 0.5% bovine serum albumin [BSA], 15 ppm ProClin, 0.05% polysorbate-20,0.25% 3-[(3-cholamidopropyl) dimethylammonio]-1-propanesulfonate [CHAPS], 5 mM ethylenediaminetetraacetic acid [EDTA] and 0.35 M NaCl). Test articles were used to prepare standard curves by serially diluting 2–1500 ng/mL in sample buffer. Capture and detection reagents were applied at 100 **μ**g/mL of biotin-conjugated goat anti-human IgG (HC + LC, monkey adsorbed, Bethyl) in PBS/0.01% polysorbate-20/0.02% NaN3 and Alexa647-anti-CDR (clone 242, Genentech) at 25 nM in Rexxip F (Gyros). The assay was run on a Gyrolab Bioaffy 200 CD, and wash steps used PBS/0.01% polysorbate-20/0.02% NaN_3_ followed by Gyros pH 11 wash buffer. The instrument was run and data analyzed as described by the manufacturer with a 1% PMT setting. The concentrations of test articles were determined from a five-parameter fit of their respective standard curves. The minimum quantifiable concentration was 8.24 ng/mL in cyno vitreous humor.

Following determination of test article concentrations in vitreous humor, pharmacokinetic parameters were determined by non-compartmental analysis using Phoenix WinNonlin (Certara Inc.).

## Supporting information

S1 Fig MALDI traces. **A.** JenKem 8-arm PEG demonstrates heterogeneity in polymer mixture and **B.** HM-MALDI trace of purified 8X Fab + 8-arm PEG.
(TIF)

S2 Fig Comparison of mass contribution from either PEG or Fab to gains in R_H_. The first data set is comprised of measured R_H_ values for 1X Fab + 1-arm PEG (purple). In this set, the mass of PEG varies, while the maleimide to Fab ratio is held constant. The second set is comprised of all the conjugates containing a 40 kDa PEG (green). In this set, the mass of the PEG is held constant while the maleimide to Fab ratio is varied. While plotting each data set revealed a linear relationship between R_H_ and MW for both data sets, linear regressions of each set revealed differences in slopes (0.11 when varying PEG mass versus 0.01 when varying Fab mass), highlighting the differences in R_H_ gains between the two sets. Though each set is limited, the extrapolated relationships suggest that PEG exercises a greater influence on R_H_ than does Fab.
(TIF)

S3 Fig Comparison of 8X Fab + 8-arm PEG conjugates demonstrates reproducibility, independent of Fab identity. **A.** UV traces, with measured R_H_ across each peak overlaid **B.** SDS-PAGE comparison.
(TIF)

S4 Fig CEX separation enabled further enrichment of desired product. **A.** Additional purification with very shallow gradient and fine fractionation was done to further enrich for desired product. **B.** Fractions were run on SDS-PAGE.
(TIF)

S5 Fig Representative SD OCT image for eye treated with 11.8 mg/eye 8X Fab + 8-arm PEG. Test article was observed within the vitreous with OCT, producing a shadow over the retinal surface **A.** Shadow over retina and test article above the retinal surface on Day 1 (OD). **B.** Shadow over retina and test article above the retinal surface on Day 14 (OD). **C.** Diffuse test article on or near inner limiting membrane, superiorly on Day 28 (OD).
(TIF)

S6 Fig Histologic findings. **A.** For eye treated with 11.8 mg/eye 8X Fab + 8-arm PEG, findings consisted of minimal to mild infiltrates of mononuclear inflammatory cells into single or multiple ocular tissues. **B.** Eye treated with 8-arm capped PEG. The character and location of mononuclear cell infiltrates was similar between animals treated with 8X Fab+8-arm PEG and those treated with 8-arm PEG alone.
(TIF)
