# Supplementary figures and images for: Identification and characterization of an octameric PEG-protein conjugate system for intravitreal long-acting delivery to the back of the eye
Source: PLoS One. 2019 Jun 28;14(6):e0218613. doi: 10.1371/journal.pone.0218613 (PMC6599134; doi:10.1371/journal.pone.0218613)

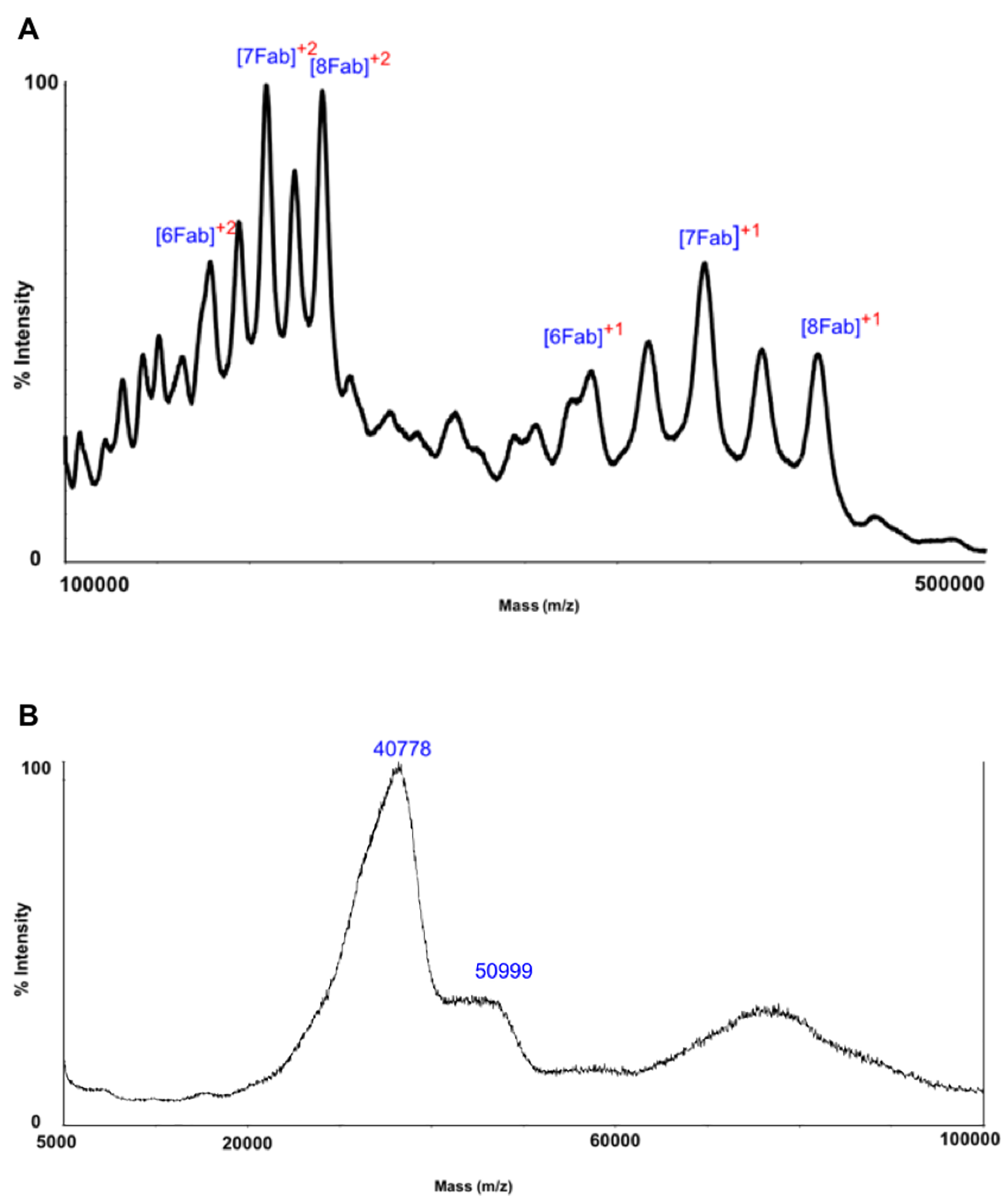

Supplement: S1 Fig — A. JenKem 8-arm PEG demonstrates heterogeneity in polymer mixture and B. HM-MALDI trace of purified 8X Fab + 8-arm PEG. (TIF) [file pone.0218613.s001.tif]

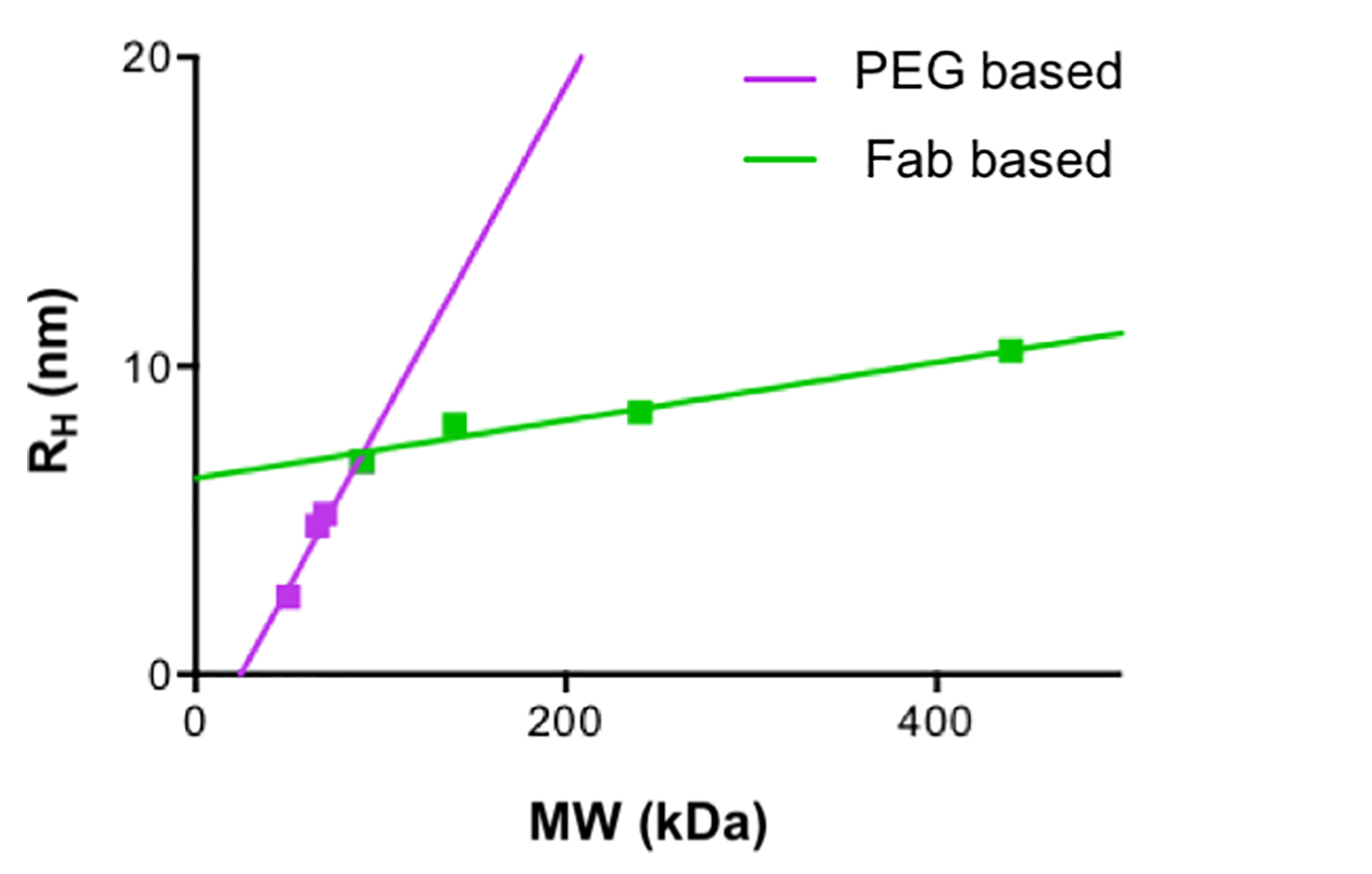

Supplement: S2 Fig — The first data set is comprised of measured RH values for 1X Fab + 1-arm PEG (purple). In this set, the mass of PEG varies, while the maleimide to Fab ratio is held constant. The second set is comprised of all the conjugates containing a 40 kDa PEG (green). In this set, the mass of the PEG is held constant while the maleimide to Fab ratio is varied. While plotting each data set revealed a linear relationship between RH and MW for both data sets, linear regressions of each set revealed differences in slopes (0.11 when varying PEG mass versus 0.01 when varying Fab mass), highlighting the differences in RH gains between the two sets. Though each set is limited, the extrapolated relationships suggest that PEG exercises a greater influence on RH than does Fab. (TIF) [file pone.0218613.s002.tif]

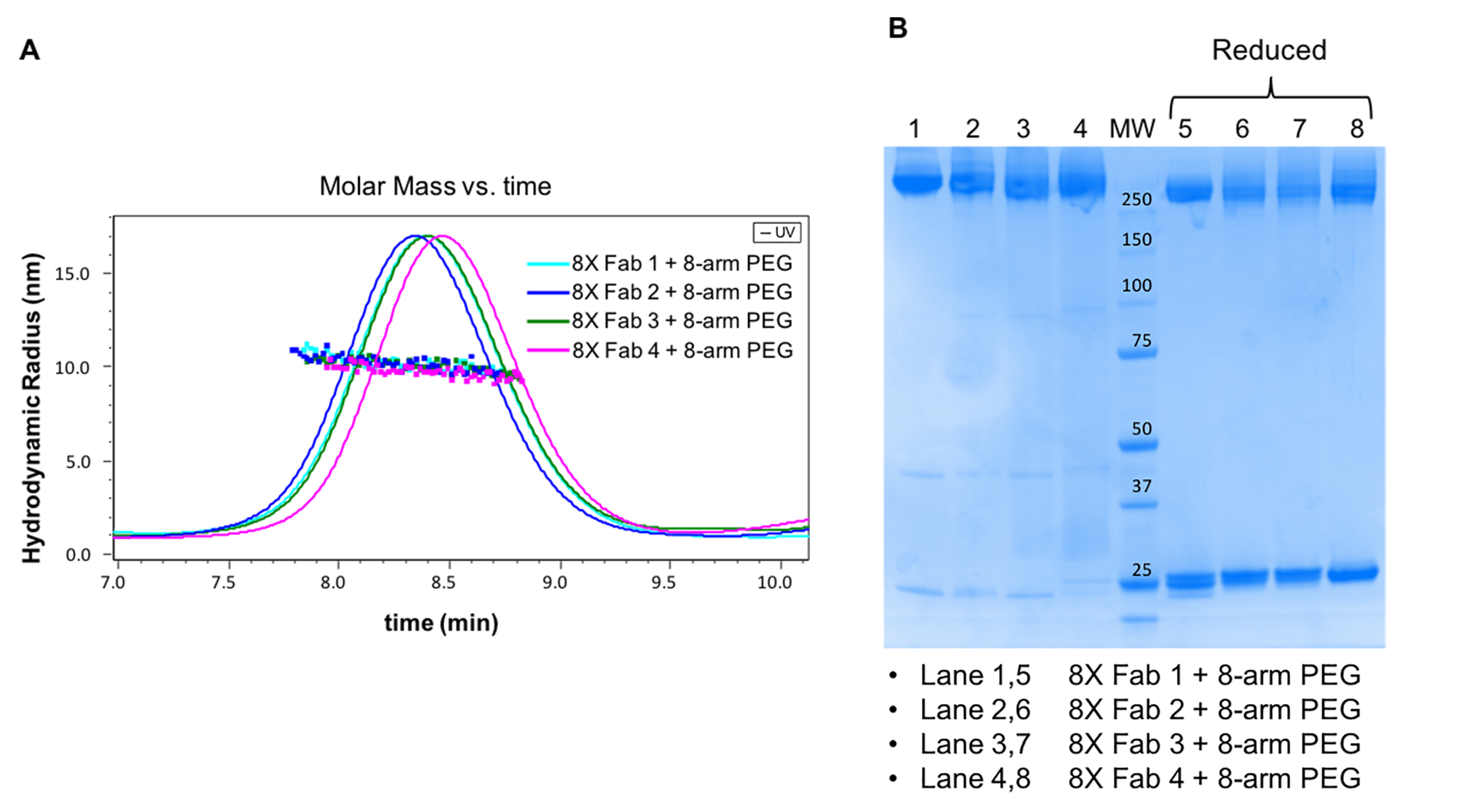

Supplement: S3 Fig — A. UV traces, with measured RH across each peak overlaid B. SDS-PAGE comparison. (TIF) [file pone.0218613.s003.tif]

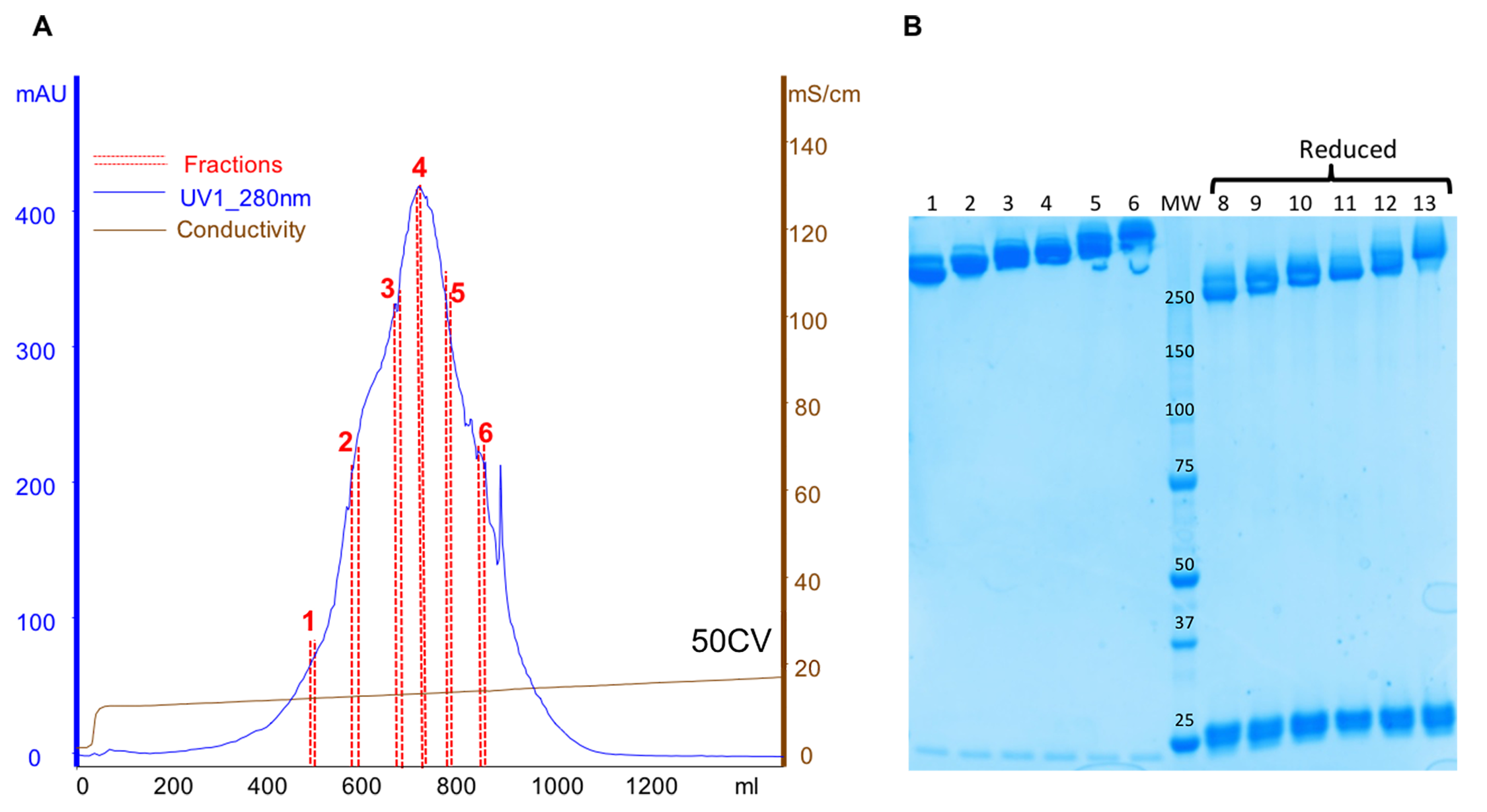

Supplement: S4 Fig — A. Additional purification with very shallow gradient and fine fractionation was done to further enrich for desired product. B. Fractions were run on SDS-PAGE. (TIF) [file pone.0218613.s004.tif]

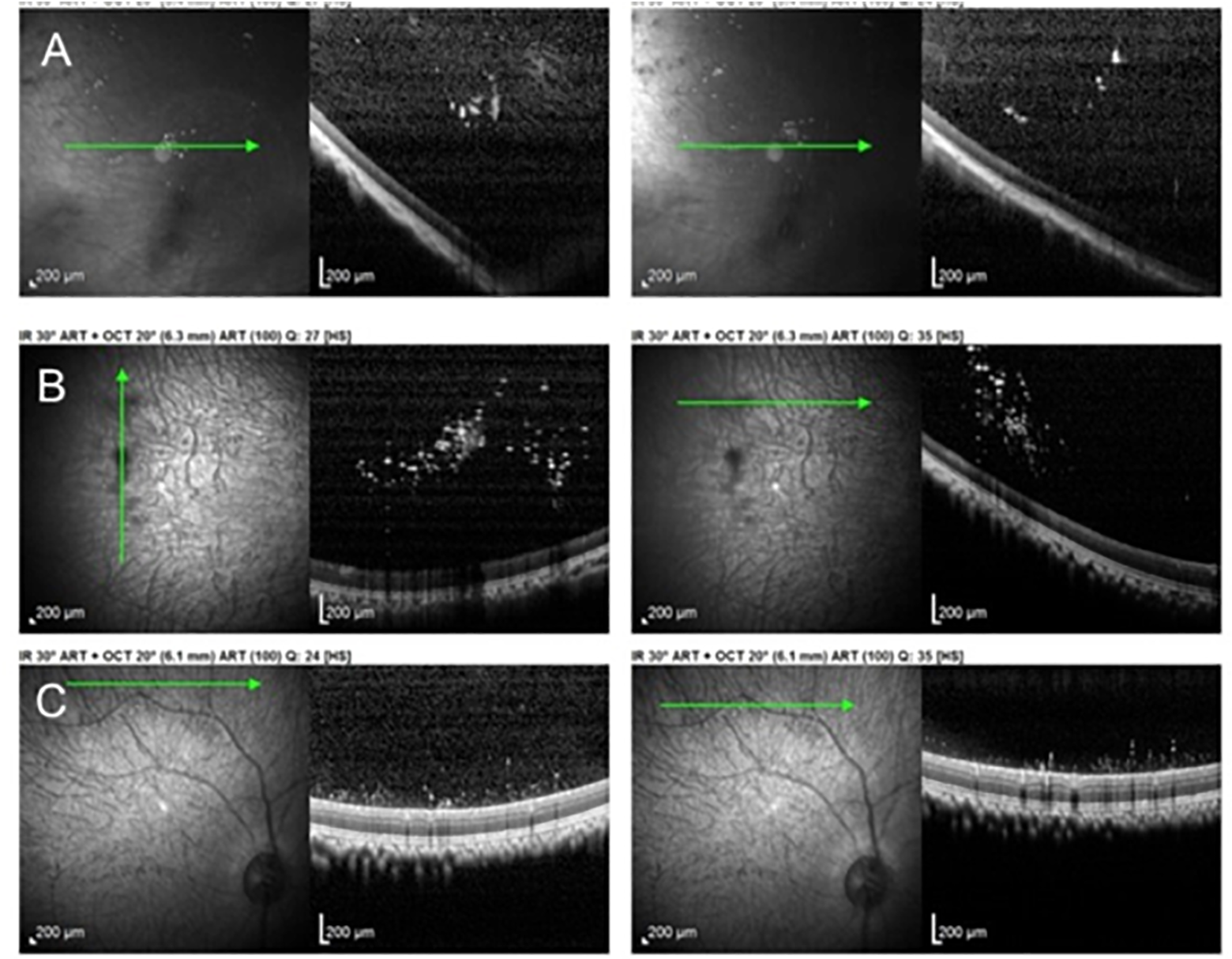

Supplement: S5 Fig — Test article was observed within the vitreous with OCT, producing a shadow over the retinal surface A. Shadow over retina and test article above the retinal surface on Day 1 (OD). B. Shadow over retina and test article above the retinal surface on Day 14 (OD). C. Diffuse test article on or near inner limiting membrane, superiorly on Day 28 (OD). (TIF) [file pone.0218613.s005.tif]

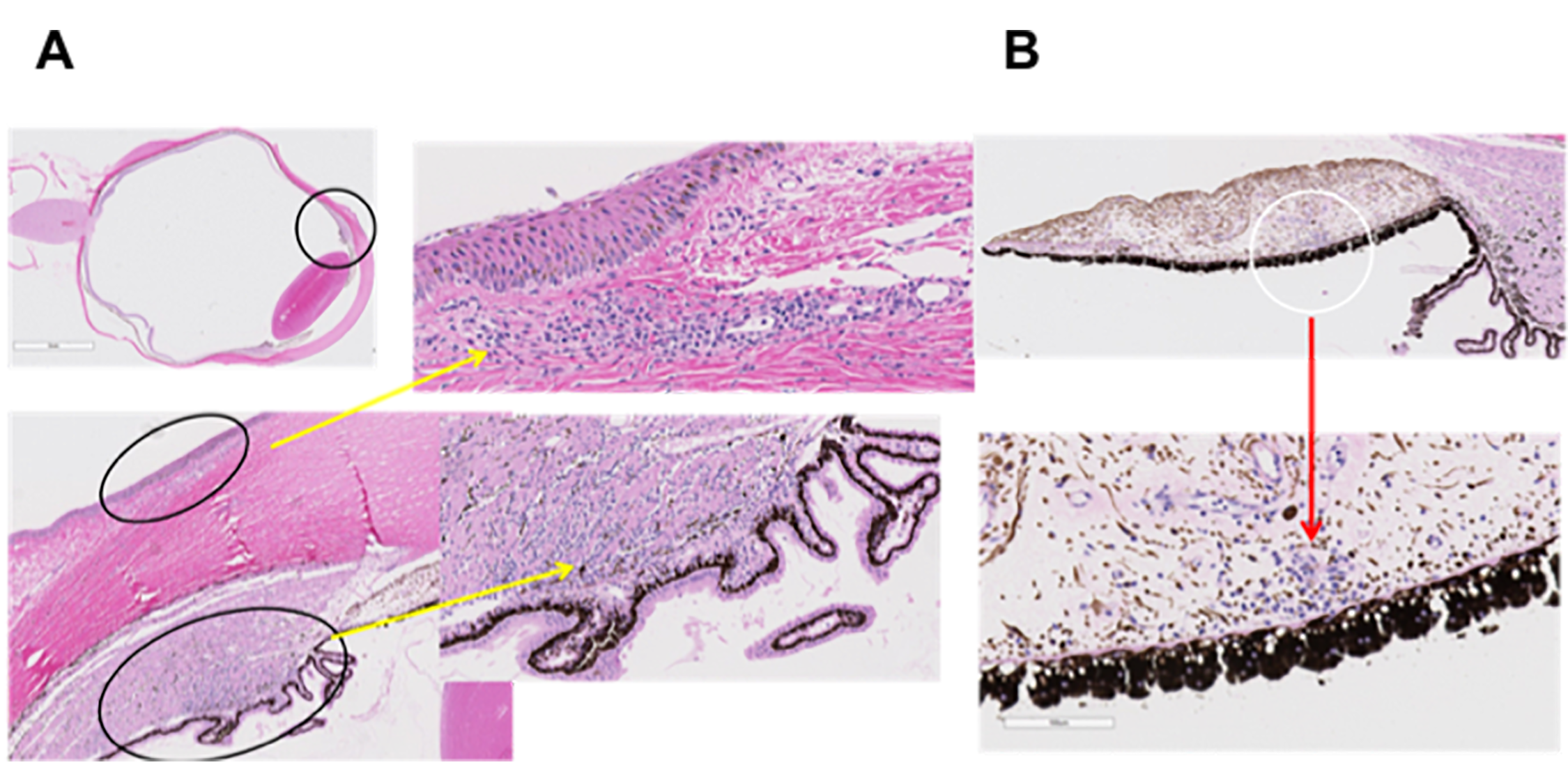

Supplement: S6 Fig — A. For eye treated with 11.8 mg/eye 8X Fab + 8-arm PEG, findings consisted of minimal to mild infiltrates of mononuclear inflammatory cells into single or multiple ocular tissues. B. Eye treated with 8-arm capped PEG. The character and location of mononuclear cell infiltrates was similar between animals treated with 8X Fab+8-arm PEG and those treated with 8-arm PEG alone. (TIF) [file pone.0218613.s006.tif]
